# Supplementary material for: Plasma Metabolomics Profile of “Insulin Sensitive” Male Hypogonadism after Testosterone Replacement Therapy
Source: Int J Mol Sci. 2022 Feb 8;23(3):1916. doi: 10.3390/ijms23031916 (PMC8836772; doi:10.3390/ijms23031916)
Supplement: Supplementary file 1 [file ijms-23-01916-s001.zip › ijms-1568324-supplementary.pdf]

Table S1. List of metabolites selected to evaluate the effect of testosterone therapy and their percentage increase or decrease before and after treatment. The values are reported as percentual difference compared to control (not included in table). *Green*, increased molecules; *red* decreased molecules.

Molecule	M.W.	Hypog. (%)	Post testost. (%)
D-Glucose 6-Phosphate (260,14)	260,14	100	105
beta-D-Fructose 1,6-bisphosphate (340.114)	340.114	350	300
D-Glyceraldehyde 3-phosphate (170,06)	170,06	5	10
Phosphoenolpyruvate (168,04)	168,04	-1	20
Lactate (90,08)	90,08	10	280
D-Glucono-1,5-lactone (178,14)	178,14	70	30
D-Ribulose 5-phosphate (230,11)	230,11	60	15
D-Erythrose 4-phosphate (200,084)	200,084	-20	-98,9
6-P-D-Gluconate (276,135)	276,135	90	10
Sedoheptulose 1,7-bisphosphate (370.14)	370.14	100	-75
NADH (663,43)	663,43	280	80
NAD (663,43)	663,43	10	-20
Glutathione disulfide (610.6)	610.6	98	-90
Glycerol-3-Phosphate (172,074)	172,074	-40	20
Dyhydroxyacetone-3P (170,06)	170,06	5	-30
Mevalonate (148,16)	148,16	290	700
Acetyl-CoA (809,57)	809,57	-48	20
Acetyl-carnitine (203,236)	203,236	-20	-90
Citrate (192,124)	192,124	-99,6	-99,55
Oxaloacetate (132,07)	132,07	-18	-1
Malate (134,0874)	134,0874	300	25
Succinate (118,09)	118,09	10	-5
2-oxoglutarato (146,11)	146,11	4	1
Oxalosuccinate (190.11)	190.11	5	-15
cis-Aconitate (174,108)	174,108	-20	-100
Glutamate (147,13)	147,13	-48	90
Glutamine (146,14)	146,14	-5	2
AMP (347,2212)	347,2212	-40	-20
ATP (507,18)	507,18	-1	-60
Aspartate (133,11)	133,11	-60	-55
Leucine/isoleucine (131,17)	131,17	-15	-47
Valine (117,15)	117,15	-15	-40
Tyrosine (181,19)	181,19	-1	-48
Phenylanine (165,19)	165,19	-5	-48
Cysteine (121,16)	121,16	200	-85
Tryptophan (204,23)	204,23	-2	-50
Methionine (149,21)	149,21	-1	-25

Alanine (89,09)	89,09	280	-55
Serine (105,09)	105,09	299	60
Threonine (119,1192)	119,1192	-5	50
Asparagine (132,12)	132,12	-97	-90
L-Arginine (174,2)	174,2	60	200
Proline (115,13)	115,13	50	47
L-Lysine (146,19)	146,19	50	25
Histidine (155,1546)	155,1546	-99	110
Uracil (112,09)	112,09	-48	-40
Carnosine (226,3)	226,3	-55	-18
